# Supplementary material for: Chaperone BiP controls ER stress sensor Ire1 through interactions with its oligomers
Source: Life Sci Alliance. 2024 Aug 5;7(10):e202402702. doi: 10.26508/lsa.202402702 (PMC11300964; doi:10.26508/lsa.202402702)
Supplement: Supplementary file 1 [file LSA-2024-02702_TableS1.docx]

| **Sequence motif** | **Forward Score** | **Backward Score** | **Max Score** | **Confirmed (NMR)** |
| --- | --- | --- | --- | --- |
| **AVVPRGS**  ^305^AVVPRGS^311^ | 0.9517 | 0.6762 | 0.9517 | **No** |
| **GSTLPLLE**  ^310^GSTLPLL^316^  ^311^STLPLLE^317^ | 0.3335  0.949 | 0.8632  0.9509 | 0.8632  0.9509 | **Yes** |
| **RNYWLLI**  ^356^RNYWLLI^362^ | 0.9427 | 0.943 | 0.943 | **Yes** |
| **KHRENVI**  ^385^KHRENVI^391^ | 0.3792 | 0.8463 | 0.8463 | **No** |
| **ENVIPADS**  ^388^ENVIPAD^394^  ^389^NVIPADS^395^ | 0.8071  0.8287 | 0.7215  0.2751 | 0.8071  0.8287 | **No** |
| **KDMATIIL**  ^442^KDMATII^448^  ^443^DMATIIL^449^ | 0.5798  0.3383 | 0.7999  0.9265 | 0.7999  0.9265 | **No** |
