# Supplementary material for: Chaperone BiP controls ER stress sensor Ire1 through interactions with its oligomers
Source: Life Sci Alliance. 2024 Aug 5;7(10):e202402702. doi: 10.26508/lsa.202402702 (PMC11300964; doi:10.26508/lsa.202402702)
Supplement: Supplementary file 2 [file LSA-2024-02702_TableS2.docx]

| Organism | UniProt ID | Sequence motif | Forward score | Backward score | Max score |
| --- | --- | --- | --- | --- | --- |
| **^310^GSTLPLL^316^** | | | | | |
| **Ire1α** | | | | | |
| Human | O75460 | GSTLPLL | 0.3335 | 0.8632 | 0.8632 |
| Tortoise | A0A8C4Y7H0 | GRAIPLL | 0.599 | 0.9006 | 0.9006 |
| Bird | A0A8C9NL08 | GSAIPLL | 0.2908 | 0.9152 | 0.9152 |
| Frog | A0A8J0TUC3 | GRAIPLL | 0.599 | 0.9006 | 0.9006 |
| Fish | A0A3B3RS82 | GSTFPLL | 0.3335 | 0.8632 | 0.8632 |
| Fish | A0A4W4DU83 | GSTFPML | 0.3528 | 0.7572 | 0.7572 |
|  |  |  |  |  |  |
| **Ire1β** | | | | | |
| Human | Q76MJ5 | GLTLAPA | 0.8926 | 0.5888 | 0.8926 |
| Birth | A0A8C4K344 | GITLARI | 0.9279 | 0.757 | 0.9279 |
| Turtle | A0A8C3F3F0 | GITLARI | 0.9279 | 0.757 | 0.9279 |
| Toad | A0A8C5Q421 | GITLAQV | 0.9441 | 0.8477 | 0.9441 |
| Fish | A0A6P7N497 | GLTLARI | 0.9152 | 0.7596 | 0.9152 |
| **^356^RNYWLLI^362^** | | | | | |
| Human | O75460 | RNYWLLI | 0.9427 | 0.943 | 0.943 |
| Tortoise | A0A8C0GAW6 | RNHWLLI | 0.8083 | 0.6218 | 0.8083 |
| Alligator | A0A3Q0H0Z4 | HNQWLLI | 0.9346 | 0.9172 | 0.9346 |
| Toad | A0A8C5R512 | RNQWLLI | 0.9346 | 0.9172 | 0.9346 |
| Fish | A0A3Q3JXF9 | RNYLLLI | 0.9427 | 0.943 | 0.943 |
| Fish | A0A8C4B6J2 | KNHLLLI | 0.8083 | 0.6218 | 0.8083 |
| Fish | A0A6J2VYF1 | QNQWLLI | 0.9346 | 0.9172 | 0.9346 |
